# Treatment of Deciduous Teeth

**Published:** 1879-02

**Authors:** W. E. Royce


### ARTICLE II.

### *Treatment of Deciduous Teeth.*

BY W. E. ROYCE, D. D. S.

Read before the Seventh and Eighth District Dental Societies of New York, October 30th, 1878.

The first imperative rule for the preservation of the teeth is absolute cleanliness. This rule cannot be put into force too soon.

We have only to glance at the "children of nature" who inhabit our Western prairies, to see that man is not by nature neat. Slovenliness, like sin, is a part of man's birthright; but by proper education he may be taught to abhor both. The child who cries at the use of the hair-brush will, probably, also object to the use of the tooth-brush, but, with proper training, he will soon learn that they are both not only endurable, but actually necessary to his comfort.

Long before the teeth make their appearance, the nurse should daily wash the mouth of her charge—taking a soft linen napkin, wrapping it around her finger, and after wetting it in lukewarm water, thoroughly rub the mouth, and especially the gums. Great care must be taken not to lacerate or chafe the delicate mucous membrane. If the mouth is carefully watched, the nurse will have timely warning of the coming tooth. Generally this is of small moment. The little pearl pushes its way up till it pricks the gum, and stands there so pure and white that we wonder how a fond mother can ever allow it to be neglected.

Few people realize what a child may suffer while teething. At this time the apical foramen is large and imperfectly formed, the tooth structure is soft, while the nerve that enters the tooth is large and sensitive. In this condition the tooth, by its gradual growth, begins to crowd against the gum, but, being unable to pierce it, is pushed back into its socket, and the child not only suffers inconvenience from the irritiation of the gum, but in many cases untold agony, on account of the nerve being pressed between the tooth and the alveolar process. At this stage, one stroke of the lancet is sufficient to almost instantly relieve the little sufferer from his torture. The lancet should not be used in any case until a thorough examination has been made and its use is found clearly indicated. This can be determined in part by the age of the child; but more definitely by the condition of the gum at the point where the tooth is supposed to be. The gum will be found more or less swollen and congested, and the mucous membrane will have a tense or glistening appearance.

When the deciduous teeth have been erupted, they are almost universally neglected. If the careful operator suggests that such teeth demand attention, he is stared at in surprise, told that it is only a "baby tooth," and asked what possible difference it can make whether the teeth drop out, or are extracted a few months earlier. Here, again, we must teach our patients; make them realize that "baby teeth" are placed in the mouths of babies for an object; until that object is accomplished they are of as much importance as any other organ, and that when their work is done, nature will remove them.

Prominent among the evils attending premature extraction of the temporary teeth may be mentioned dyspepsia, contracted-arch, irregularity and consequent loss of the permanent teeth. We daily see children whose teeth have reached such a condition that mastication is an impossibility. In consequence of this, the food is passed to the stomach not only without mastication, but also without insalivation. The stomach, called upon to perform this excessive and unnatural work, soon gives out. The saliva becomes acid, and the permanent teeth, when erupted, bathed in this abnormal fluid, soon decay. May not the premature loss of the deciduous teeth be regarded in *very many* cases as the primary cause of dyspepsia ?

It is held by some that the alveolar arch is not absolutely contracted by the early loss of the temporary teeth, but, from want of use, it ceases to expand. Be this as it may, the result is the same. When the permanent teeth make their appearance, the arch is not large enough to receive them and they are consequently crowded from their proper position. The treatment of irregularity is discussed at length in our text-books, but the first rule is to remove, or —more properly in this case—avoid creating, the cause.

As the permanent teeth, lodged in their respective sacks, are gradually developed, and push their way out through the alveolar process, the roots of the temporary teeth are absorbed, until at last, when scarcely more than the crown remains, they drop out and give place to the permanent ones. The absorption of the root of the temporary tooth depends upon the life of the pulp, and ceases, if by any means that life is destroyed. In such a case, the permanent tooth, finding its proper course obstructed, seeks another, and is often erupted high up on the labial or palatine wall of the process. Or it may crowd directly upon the unabsorbed root, producing the most intense pain. Here extraction of the deciduous tooth is indicated. Alveolar abscess associated with a deciduous tooth should always be looked upon with concern, and unless it speedily responds to medication, should be regarded a sufficient cause for the extraction of the offending tooth.

Remembering the importance of not only preserving the deciduous teeth, but also of preserving them alive, let us examine their structure.

We find the enamel *thin*—affording but a slight protection to the tooth. The texture of the dentine is less than that found in the permanent teeth, while the pulp cavity is much larger in proportion to the size of the tooth ; all indicating their quick destruction if attacked by caries.

It has long been conceded that the proper treatment for caries is to remove the decomposed tooth structure and fill the cavity. Here our *definite* instruction suddenly stops. One tells us *never* to use anything as a filling material but gold. Another says use nothing but plastic fillings. This discussion has continued for years and will continue for years to come. It would appear, however, that the discussion must be confined to the filling of permanent teeth, as there can be no question in regard to which is preferable in temporary teeth.

In a very great majority of cases, gold is the best material for filling permanent teeth, but it is hard to conceive of an instance where its use would be admissible in filling deciduous teeth. If the patient is very young, he has not the strength to endure the protracted operation of filling with gold. In fact, it is doubtful whether any child can be subjected to the fatigue attending such an operation, without injury.

As the strength of the child increases, the strength of the tooth decreases. So at the age of eight or ten, even if the strength of the child is sufficient for the operation, the root of the tooth is so far absorbed that to apply the force necessary to condense a gold filling would be almost certain to loosen the tooth.

One of the chief qualities to recommend gold is its durability: a quality almost superfluous in a material for filling temporary teeth. Tin is not as objectionable as gold, as it is softer and more easily worked. Still, the force required to condense it is greater than can, with safety, be applied to a temporary tooth.

Plastic materials, although easy to insert, are often objectionable on account of the nature of their ingredients.

As the root of the tooth is absorbed the pulp also disappears, and the tooth is compelled, to a great extent, to depend upon the surrounding soft parts for nutrition. Or, in other words, nutrition is accomplished by an endosmotic and exosmotic action. With this fact in mind, and remembering the lack of density found in the deciduous dentine, it can be seen how readily any injurious substance, placed in such a tooth, would be conducted to the general system. Amalgam, on account of its mercury would be objectionable in a tooth where the root was absorbed to any extent. It may be used, however, where a very hard filling is demanded —as upon the grinding surface of a molar—and the patient is so young that absorption has but slightly progressed.

Oxy-chloride of zinc possesses few qualities to recommend it in these cases, and should be avoided in partially absorbed teeth. Gutta-percha is undoubtedly the best material in all cases where hardness is not required ; as on approximate, buccal, and palatine surfaces. Hill's Stopping, composed of gutta-percha, quick-lime, quartz and felspar, substances which cannot injure the system if absorbed, may be used where a harder material is required.

It is true that Hill's Stopping is not a permanent filling, but the teeth of which we are speaking have but a very short time to remain in the mouth, and even if the filling has to be replaced a few times, it is much better than to endanger the health of the patient.

That which has been said in regard to absorption into the system of materials used in filling, applies with still more force in regard to materials used in treating temporary teeth. As an example, arsenious acid should never be used in the deciduous teeth. If the pulp is exposed to such an extent that it is necessary to destroy its vitality (the importance of avoiding such a condition we have already seen,) the devitalization may be accomplished without danger either to the system or surrounding parts, by the use of a crystal of carbolic acid.

As a rule, mouth-washes and dentifrices should be avoided. In the mouths of some children, however, in spite of a thorough use of the tooth-brush, there is deposited upon the labial surface of the oral teeth a dark brown or green stain, which is supposed to be a deposit from the mucous. When this secretion is in a more acid condition than is normal, here a dentifrice of finely levigated chalk, together with a mouth-wash of lime water, will be found advantageous, both in removing the stain by friction and in restoring the fluids of the mouth to a normal condition by their alkaline properties.

Chalk and lime-water are also recommended where there is a surplus of organic matter in the teeth. The chalk is placed in contact with the teeth, upon retiring, and allowed to remain through the night. Lime-water is used as a mouth-wash. The theory is, that a part of the lime and chalk is absorbed directly into the teeth. It seems more probable, that the substances being placed in the mouth, pass along the alimentary canal, are taken up by the absorbents, and eventually reach the teeth by means of the general circulation.

The result would be the same in either action, namely, to harden these teeth so that they may perform perfectly their work, which, although short, is of the greatest importance.—*Dental Advertiser.*

---

